# Development of biobanking infrastructure in Armenia: opportunities and challenges

**DOI:** 10.3389/fpubh.2025.1749588

**Published:** 2026-01-13

**Authors:** Gohar Mkrtchyan, Kristina Avanesyan, Hovsep Ghazaryan, Merry Mazmanian, Marie-Alexandra Alyanakian, Arsene Mekinian, Arsen Arakelyan

**Affiliations:** 1 Institute of Molecular Biology NAS RA, Yerevan, Armenia; 2 Santé Arménie French-Armenian Health Foundation, Yerevan, Armenia; 3 Necker Biological Resources Center and Immunology Laboratory, Necker Hospital for Sick Children, AP-HP, Paris, France; 4 Department of Internal Medicine, Sorbonne University, DMU i3D, Saint-Antoine Hospital, AP-HP, Paris, France

**Keywords:** Armenia, biobanking, disease-oriented biobank, international collaboration, research infrastructure

## Abstract

Biobanking has become a cornerstone of biomedical research and precision medicine, enabling population studies, biomarker discovery, and translational research. However, while high-income countries operate mature biobanking systems, middle-income and transitional economies encounter distinct barriers to establishing sustainable and internationally compatible infrastructures. This mini review examines the development of biobanking in Armenia as a representative middle-income case. It highlights key opportunities, such as strong diaspora scientific networks, a growing biotechnology sector, increasing national interest in precision medicine, and Armenia’s strategic geographic position between Europe and Asia. At the same time, significant challenges persist, including limited funding, regulatory and ethical gaps, infrastructure constraints, shortages in specialized technical personnel, and socio-cultural barriers related to sample donation and data sharing. The review outlines strategic approaches adopted in Armenia, including international collaboration, capacity-building initiatives, and alignment with global best practices. Lessons from Armenia’s experience may guide other middle-income and post-Soviet countries in developing effective and resilient biobanking systems.

## Introduction

Biobanks are structured repositories that store high-quality biological specimens along with associated clinical and demographic data. They serve as critical infrastructure for advancing biomedical research, enabling the study of genetic, environmental, and lifestyle factors influencing health and disease (1). Today, the reliability of research findings is closely tied to the quality of biospecimens, and inconsistent or contradictory results are often attributable to poor sample quality.

The global impact of biobanking is well documented, with large-scale initiatives in high-income countries, such as the UK Biobank and the Estonian Biobank, demonstrating their value in elucidating disease mechanisms, identifying novel biomarkers, and supporting targeted therapeutic development (2). These sophisticated networks, often integrated with national healthcare systems, illustrate how biobanks can drive scientific innovation and improve clinical outcomes. The field has experienced remarkable growth, with the number of biobanks worldwide increasing from approximately 300 in 2000 to over 2,000 by 2020, representing a significant research infrastructure investment globally (3, 4).

Biobanks vary widely in scale and purpose, ranging from small, investigator-initiated collections to extensive national efforts. These include population biobanks, disease-oriented collections, clinical biobanks embedded within healthcare institutions, and project-specific repositories created for time-limited studies. This typological diversity reflects differences in research priorities, healthcare systems, and resource availability across countries (5).

The COVID-19 pandemic has highlighted the critical role of biobanks in supporting public health, from disease surveillance to diagnostic validation and vaccine development (6). However, in many middle- and low-income countries (LMICs), biobanking remains an emerging field, and biobank numbers remain insufficient given the population and clinical needs. Although biobanks in some LMICs have participated in global consortia, such as the H3Africa Biobank Network, Indian National Biobank, their participation remains limited relative to the epidemiological burden (7, 8). In LMIC settings, biobanking facilities are often located within academic or research institutions and focus primarily on the genetic basis of prevalent diseases. Research laboratories frequently maintain independent sample collections, with a primary emphasis on DNA because of its relative ease of collection, processing, and long-term storage. Development is frequently constrained by limited funding, underdeveloped infrastructure, regulatory and ethical complexities, and shortages of skilled personnel, laboratory information management systems (LIMSs), and clear governance frameworks (9).

Addressing these challenges through strategic collaboration with existing infrastructures could enhance the visibility and perceived value of biobanks, helping attract sustained funding, institutional commitment, and public trust (10, 11).

### Biobanking in Armenia: context and rationale

Armenia, a small country in the South Caucasus with a population of about three million people, represents a particularly interesting case for biobank development. For Armenia, establishing a robust biobanking infrastructure represents both a significant challenge and a strategic opportunity to strengthen its healthcare system and research capabilities. Armenia, classified as a developing country by international organizations, such as the United Nations and the World Bank, illustrates the potential of a transitional economy. Since gaining independence from the Soviet Union in 1991, Armenia has undergone significant political and economic transformation. Classified as an upper-middle-income country, Armenia remains a transitional economy facing typical middle-income challenges, poverty (26.4%), unemployment (18.1%), and regional instability, while demonstrating notable strengths in education, technological capacity building, and healthcare reform (12).

Importantly, Armenia possesses a strong legacy in biomedical sciences, particularly in Molecular Biology and Genetics, dating back to the Soviet era. Research institutions established during this period have maintained their scientific capabilities despite resource limitations (13). Today, the country invests only 0.32% of its GDP in research and development, significantly below the OECD average of 2.5%, but has targeted biomedical sciences as a priority area. Notably, over the long term, Armenia’s R&D expenditure as a share of GDP has not exceeded 0.18% (14).

The Armenian population represents a genetic isolate with unique genomic features resulting from historical founder effects and limited admixture (15). Genetic studies have revealed distinctive patterns in mitochondrial DNA haplogroup distribution, particularly haplogroup U7 (16), and population-specific variant frequencies relevant to disease risk assessment (17, 18). This genetic homogeneity, together with well-documented genealogical records in many communities, provides valuable opportunities to study genetic factors underlying disease susceptibility and progression. Armenia’s disease burden reflects both demographic transitions and socioeconomic challenges, with cardiovascular diseases accounting for approximately 53% of mortality and cancer contributing around 20% (19). These epidemiological patterns create specific research priorities that a well-designed biobank can address through targeted collections and analysis.

Despite these advantages, biobanking initiatives in Armenia have been limited by resource constraints and structural challenges within the research ecosystem. The country’s research infrastructure was significantly affected by the collapse of the Soviet Union, with subsequent economic difficulties hampering the development of modern scientific facilities. Nevertheless, recent years have witnessed renewed efforts to strengthen the nation’s biomedical research capabilities, supported by international collaborations and diaspora networks.

This paper describes the establishment of the Santé Arménie Biobank, a disease-oriented biobank developed in collaboration with the Franco-Armenian Medical Association. By analyzing infrastructural requirements, governance frameworks, ethical considerations, and international collaboration pathways, we identify the critical challenges and opportunities involved in biobank development within resource-constrained settings. This study illustrates how a country with a modest economic profile but considerable scientific potential can leverage biobanking to enhance its healthcare and research systems.

### Santé Arménie Biobank: vision and implementation

The Santé Arménie Biobank was established in 2023 by the French-Armenian Health Foundation Santé Arménie (20) in collaboration with the Institute of Molecular Biology of the National Academy of Sciences of the Republic of Armenia (NAS RA). Its primary goal is to collect and store biological specimens and clinical data from patients affected by regionally prevalent diseases, thereby creating an infrastructure for biomedical research and precision medicine in Armenia.

Implementation began with a feasibility study to assess local capacity, stakeholder readiness, and legal requirements. Institutional support was secured from the French-Armenian Academic Research Organization (FAARO) in collaboration with local hospitals. FAARO’s mandate is to advance scientific medical research in Armenia, address public health challenges, strengthen international collaboration, and improve access to modern treatments.

Standard operating procedures (SOPs) for sample collection, processing, and storage were developed in line with guidelines from the International Society for Biological and Environmental Repositories (21). Ethical approval was obtained from local ethics committees for each registry, with particular emphasis on informed consent. The consent process was designed to be transparent, ensuring that participants understood the scope of the research, the use of biospecimens, and their right to withdraw at any time.

Workforce training was a central component of implementation. Personnel received instruction in biospecimen handling, data entry, and patient communication, with technical assistance provided by European colleagues from Santé Arménie with expertise on biobanking. This not only ensured compliance with international standards but also contributed to local capacity building in biobanking and biomedical research.

Cold storage facilities, including ultralow-temperature freezers and liquid nitrogen systems, were installed. A customized data management platform was implemented to ensure the secure linkage of biospecimens with clinical metadata while maintaining confidentiality.

*Storage capacity and technical infrastructure:* The biobank is equipped with advanced infrastructure designed to ensure the long-term preservation, traceability, and integrity of biospecimens. A secure digital platform links each specimen to clinical and phenotypic data, maintaining confidentiality and compliance with international governance standards.

*Integration with clinical registries:* The Biobank collaborates with several national and international clinical registries to support biomedical research and precision medicine. National partnerships include *NAREG* (Armenian Nationwide Registry of Systemic Autoimmune and Autoinflammatory Diseases), coordinated by the Santé Arménie Health Foundation, and *ARMI* (Armenian National Registry of Myocardial Infarction), a nationwide observational study enrolling STEMI patients in collaboration with the Armenian Cardiologists Association. Internationally, the Biobank participates in EUSTAR (EUropean Scleroderma Trials and Research Group), the GFM (Groupe Francophone des Myélodysplasies) registry on myelodysplastic syndromes, and the JIR Cohorte (Juvenile Inflammatory Rheumatism Cohort).

To date, more than 1,600 biospecimens have been collected and securely stored. Samples are collected from Yerevan medical centers in both inpatient and outpatient settings. Physician-mediated enrolment follows registry integration frameworks, with informed consent obtained from all participants. Engagement with the medical community and patient education on the value of research and data protection have been central to achieving high participation. These collaborations enhance the Biobank’s scientific value, promote Armenia’s integration into global biomedical research, and strengthen local capacity through multidisciplinary partnerships.

*Sample processing workflow:* A standardized processing pipeline ensures biospecimen quality, reproducibility, and compliance with international standards. Following informed consent, blood samples from collaborating sites are transported under controlled conditions and processed using validated protocols. Each specimen is barcoded, digitally registered, and securely linked to clinical and demographic data. Samples are stored at appropriate temperatures with continuous monitoring to safeguard integrity. Trained personnel manage all aspects of handling, quality control, and data entry, ensuring consistency, adherence to best practices, and the development of local biobanking capacity.

### Challenges and strategic responses

The key challenges encountered during the development of the biobank included the following:

*Resource limitations:* Financial constraints represent a major barrier to sustainable biobanking in Armenia. With approximately 0.32% of GDP allocated to R&D, national research funding remains limited. Competing priorities within healthcare and research budgets, high costs of specialized equipment often requiring importation, limited commercial investment, and ongoing operational expenses further exacerbate the challenge (22). Infrastructure gaps include inconsistent power supplies in some regions; limited facilities specifically designed for biospecimen storage; transportation and logistics challenges for sample collection from rural areas; insufficient information technology infrastructure; and limited laboratory space meeting international standards (23).

*Regulatory gaps:* Armenia currently lacks specific legislation governing biobanking activities, resulting in uncertainty around sample ownership, consent requirements, and the provision of samples. Existing relevant legislation includes the Law on Scientific and Scientific-Technical Activities, the Law on Protection of Personal Data, healthcare regulations on medical specimens, intellectual property laws, and international agreements on biological material transfer. To address this regulatory gap, we have developed a comprehensive biobank governance framework aligned with international guidelines (ISBER, BBMRI-ERIC) and plans to become an institutional member of ISBER and to establish collaboration with BBMRI-ERIC. These steps will enable participation in quality assessment programs and harmonization of activities. Collaboration with the Ministry of Health of RA is planned to develop an institutional policy that could serve as a foundation for future national biobank legislation. Additionally, enhanced data protection measures are being proposed to exceed current national standards and ensure alignment with the GDPR and other international norms. The ongoing evolution of Armenia’s legal framework for biomedical research presents both challenges and opportunities, enabling the biobank to actively contribute to shaping balanced regulations that support scientific advancement while safeguarding participant rights.

*Sample turnover and sustainability:* Current infrastructure accommodates approximately 200,000 samples, representing 10 years of collection at projected rates. A phased expansion model is in place including monitoring capacity utilization and planning expansions implementing sample retirement protocols for collections with completed primary objectives after 10-year retention period, and transitioning to energy-efficient equipment and solutions, such as ThermoFisher ultra-low freezers and alternative energy sources (e.g., solar panels). Additional long-term sustainability strategies involve diversification of funding sources and continuous application for external grant support.

*Technical expertise:* Expertise limitations include a shortage of specialists trained in modern biobanking procedures, limited experience with quality management systems for biobanking, the need for bioinformatics specialists to manage biobank data, brain drain of scientific talent to higher-income countries, and limited training opportunities within Armenia for biobank-specific skills. In response, staff training programs have been initiated in the areas of biospecimen handling, quality assurance, and data management. Partnerships with international networks are also being leveraged to provide advanced training opportunities, mentorship, and capacity-building workshops within Armenia.

*Reliable power supply:* Ensuring a stable and uninterrupted electricity supply is essential for maintaining the integrity of biospecimens stored in ultralow-temperature freezers. Inconsistent power poses a critical risk of temperature excursions, which could compromise sample viability and long-term preservation.

To address this challenge, the biobank established a comprehensive and redundant power infrastructure designed to ensure the continuous operation of critical storage systems. An uninterruptible power supply (UPS) system was installed to deliver immediate short-term power during outages, thereby preventing temperature fluctuations during transitions. In parallel, diesel generators were deployed to provide sustained backup power during extended outages. Both systems are subject to regular testing and preventive maintenance in accordance with the manufacturer’s specifications to ensure optimal performance. These measures align with the best practices recommended by the ISBER and the OECD Guidelines on Human Biobanks and Genetic Research Databases, ensuring compliance with international standards for biorepository reliability and sample preservation (21, 24).

*Sociocultural barriers:* Skepticism about biomedical research driven by low health literacy and limited public awareness poses significant challenges to participant recruitment. Misconceptions about genetic studies and concerns over privacy further contributed to reluctance.

*Public awareness and engagement:* Social and cultural factors include limited public understanding of biobanking concepts, potential cultural sensitivities regarding biological sample donation, the need for transparent communication for research purposes to ensure appropriate consent processes across educational levels and the ability to build trust in research institutions and governance systems. To address these issues, awareness-raising activities have been implemented through community engagement initiatives, including monthly public health forums at community centers, participation in” Science Week” annual events and medical expos. Transparent communication of consent processes and the involvement of healthcare professionals in public dialogue have been central to these efforts. Training programs for clinicians and nurses in patient communication are being developed to improve trust-building and recruitment strategies. These combined efforts have contributed to a substantial increase in consent rates over the first year of implementation, demonstrating a measurable impact on community acceptance and participation.

### Opportunities and enabling factors

*Digital health infrastructure and biobank integration:* Armenia’s progress in digital health creates a strong foundation for biobank integration. The country has successfully developed application programming interfaces (APIs) that connect laboratory information systems with the national electronic health operator via LOINC standardized codes. This integration reduces medical errors, eliminates redundant testing, and decreases administrative burdens on healthcare providers. Security measures ensure the protection of patient data, whereas the “Armed eHealth” mobile application provides patients with direct access to their laboratory results (25).

These established digital systems foster an ideal environment for biobank development, where secure data handling, standardized coding, and digital interoperability are already operational. The existing infrastructure facilitates efficient linkages between clinical data and biobanking platforms, accelerating research, supporting precision medicine, and improving public health outcomes. Armenia’s readiness for digital health thus positions it as a regional leader in integrating biobanks into national healthcare systems.

*Diaspora networks and international collaboration:* Armenia benefits from a large global diaspora with strong scientific connections. Diaspora resources include scientific expertise in biomedical research and biobanking, potential funding through diaspora philanthropy, knowledge transfer through visiting scientist programs, facilitation of international partnerships, and advocacy for Armenian research priorities in global forums.

*Technological innovation and adaptation:* Armenia have demonstrated strengths in terms of technology adaptation that could benefit biobank development. Technology strengths include a growing information technology sector with potential applications to biobank data management, experience adapting technologies to local contexts and resource constraints, innovation in cost-effective solutions for scientific infrastructure, strong engineering education producing technical talent, and an emerging start-up ecosystem including biotech ventures.

*Strategic geographic position:* Armenia’s location provides unique opportunities. Geographic advantages include positioning between European and Asian biobanking networks, such as the European, Middle Eastern & African Society for Biopreservation and Biobanking (ESBB) and the Public Population Project in Genomics and Society (P3G), potential to serve as a regional hubs for the South Caucasus, access to diverse population groups with unique genetic characteristics, proximity to Middle Eastern and Central Asian research networks, and connections to both the EU and Eurasian Economic Union scientific communities (26).

*Privacy and data protection:* Armenia’s evolving legislative landscape presents significant opportunities for biobank development through the establishment of comprehensive data protection frameworks. The ratification of the Oviedo Convention in 2024 provides a foundational ethical framework that prioritizes human dignity while enabling scientific progress (Convention on Human Rights and Biomedicine; ETS No 164) (27). This international agreement, coupled with Armenia’s commitment to developing biobank-specific regulations, creates favorable conditions for research infrastructure growth. The implementation of robust data protection measures, including anonymization protocols, secure sharing mechanisms, and cross-border transfer provisions, establishes the necessary safeguards that facilitate both national research initiatives and international collaboration. This balanced approach to governance enables Armenian biobanks to participate in global scientific networks while maintaining high ethical standards and respecting participant rights.

## Conclusion

The establishment of a biobank represents a significant achievement in developing functional biobanking infrastructure within resource-constrained environments. This initiative demonstrates how strategic adaptation of international best practices to local contexts can create sustainable biomedical research resources that advance both national priorities and global scientific collaboration․ Our experience confirms that successful biobanking in transitional economies requires thoughtful navigation of multiple challenges, including regulatory frameworks, resource limitations, infrastructure constraints, technical expertise gaps, and sociocultural considerations (28). The adaptive approaches implemented in Armenia offer valuable insights for similar initiatives in comparable settings worldwide.

The biobank’s development strategy, informed by lessons from Estonia and Latvia, prioritized disease-specific collections relevant to local health burdens while implementing phased development with clear milestones (29). Leveraging Armenia’s geographic position between European and Asian research networks has increased its potential to emerge as a regional biomedical research hub, creating scientific opportunities beyond national boundaries.

International partnerships and diaspora engagement have helped address resource limitations, while attention to ethical standards and cultural sensitivities has supported community acceptance. Early sustainability planning, through workforce development, strong governance, and ongoing training, has strengthened long-term stability. University partnerships further enhance sustainability by integrating biobanking into curricula, offering internships, and converting trainees into permanent staff through structured career development frameworks. Oversight by the Scientific Advisory Board and Ethics Committee, guided by documented standard operating procedures and regular review cycles, ensures compliance and quality management. Recognized as a critical research infrastructure and incorporated into institutional strategic planning, the biobank establishes biobanking as an essential national capacity.

Future priorities include expanding collections, investing in analytical technologies, enhancing international collaboration, and advancing precision medicine tailored to the Armenian population. As global biobanking networks evolve, resources like the Santé Arménie Biobank will help ensure broader population representation in biomedical research.

In summary, resource constraints do not preclude meaningful participation in global biobanking. With strategic planning, capacity building, and community engagement, transitional economies can build resilient biobanking systems that support scientific progress and national health research capacity.
